# Experimental investigation on the coupled effect of effective stress and gas slippage on the permeability of shale

**DOI:** 10.1038/srep44696

**Published:** 2017-03-17

**Authors:** Diansen Yang, Wei Wang, Weizhong Chen, Shugang Wang, Xiaoqiong Wang

**Affiliations:** 1 State Key Laboratory of Geomechanics and Geotechnical Engineering, Institute of Rock and Soil Mechanics, Chinese Academy of Sciences, Wuhan, Hubei 430071, China; 2 University of Chinese Academy of Science, Beijing, 100049, China; 3 Geotechnical and Structural Engineering Research Center, Shandong University, Jinan, Shandong, China; 4 Unconventional Natural Gas Institute, China University of Petroleum, Beijing 102249, China

## Abstract

Permeability is one of the most important parameters to evaluate gas production in shale reservoirs. Because shale permeability is extremely low, gas is often used in the laboratory to measure permeability. However, the measured apparent gas permeability is higher than the intrinsic permeability due to the gas slippage effect, which could be even more dominant for materials with nanopores. Increasing gas pressure during tests reduces gas slippage effect, but it also decreases the effective stress which in turn influences the permeability. The coupled effect of gas slippage and effective stress on shale permeability remains unclear. Here we perform laboratory experiments on Longmaxi shale specimens to explore the coupled effect. We use the pressure transient method to measure permeability under different stress and pressure conditions. Our results reveal that the apparent measured permeability is controlled by these two competing effects. With increasing gas pressure, there exists a pressure threshold at which the dominant effect on permeability switches from gas slippage to effective stress. Based on the Klinkenberg model, we propose a new conceptual model that incorporates both competing effects. Combining microstructure analysis, we further discuss the roles of stress, gas pressure and water contents on gas permeability of shale.

With recent development of novel technologies, unconventional gas extraction has made great success in the North America. Shale gas has been considered as an attractive low-carbon solution for the transition period to a future power by renewable energy^1^. As an important supplement to conventional energy resources, shale gas has obtained an increasing attention in the past years. Due to its extremely low permeability, shale gas is still difficult to be recovered, particularly in deep formations. For example, the average burial depth of shale gas in the US is about 800–2000 m, while the average depth in China is over 3500 m where the stress is significantly high (>50 MPa)^2^. In order to better evaluate the production of shale gas, an accurate permeability of shale under *in situ* stress conditions is required. As the mean pore size of shale is often at the nanoscale, the permeability of shale is substantially low, typically less than 10^−19^ m^2^, which is difficult to be measured in the laboratory. Inert gases such as helium, azote, nitrogen, are often used to measure the low permeability. Gas can shorten the testing time and can also avoid the hydration reaction of shale with high content of clay minerals that are sensitive to water. However, gas slippage effect could be very large in nanopores and thus could affect gas permeability measurement. Numerous investigations have been performed to study gas slippage phenomenon which was initially noted in the 1940s^3^,^4^,^5^,^6^,^7^,^8^. Due to gas slippage, the steady-state flow rate through small capillaries is higher, which results in an overestimation of permeability. To estimate the gas slippage effect on the permeability, Klinkenberg proposed an empirical equation written as


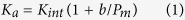


where *K*_*a*_ is the apparent permeability, *K*_*int*_ is the intrinsic permeability, *P*_*m*_ is the average gas pressure in pores, and *b* is the slope of Klinkenberg straight line related to the mean free path of gas molecules. Many researchers have investigated this phenomenon from the flow dynamics of gases in micro-channels with high Knudsen numbers (*K*_*n*_), defined as the ratio of the molecular mean free path to the characteristic length of the flow path^9^,^10^. Methods such as the direct simulation Monte Carlo approach and the lattice Boltzmann method have been used to estimate the gas slippage effect^3^. A few new expanded models have been proposed to correct Klinkenberg’s equation^4^,^6^. In the experimental tests, Klinkenberg effect is often characterized using different gas pressures to measure permeability^11^. High gas pressure is often applied to reduce the gas slippage effect because high gas pressure can lead to a smaller mean free path of gas molecules and thus minimize Klinkenberg effect. However, high gas pressure also reduces the effective stress and thus increases permeability. The dependence of permeability on the stress has been well observed in the laboratory. Dong *et al*. in 2010 found that the permeability of shale was more sensitive to the effective confining stress than sandstone^12^. Based on numerous experimental data, stress dependent permeability could be described by either an exponential relationship^13^,^14^ or a power law^15^,^16^,^17^,^18^. Katsuki *et al*. in 2013 discussed poroelastic effects on the stress-dependent permeability of a stiff shale and found that pore pressure changed gas permeability of shale significantly^19^. However, there have been only a few studies to date aiming at understanding the coupled effect of gas slippage and effective stress principle on the permeability of shale. This paper will present an experimental investigation on this topic and discuss various factors influencing the gas permeability of shale.

## Methods

### Pressure transient method and experimental setup

**Figure 1:**
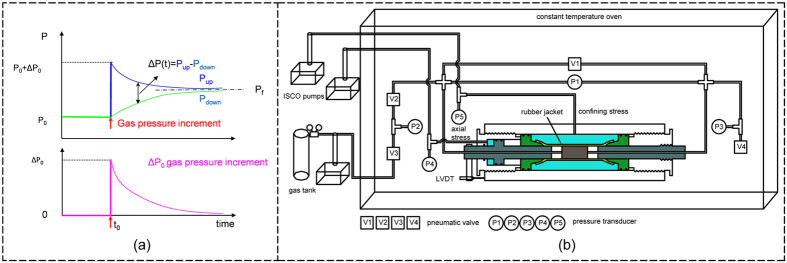
Illustration of the pressure transient method for gas permeability measurement (**a**) principle of the method; (**b**) the experimental setup).

Pressure transient method or known as pressure pulse decay^13^,^20^,^21^ is often used to effectively measure low permeability of tight rocks (<100 nD (10^−19^ m^2^)), as it is less time consuming. Recently, pressure transient measurements on ultra-tight rocks have been interpreted by accounting for the effects of gas-slippage^22^, transitional flow, and Knudsen diffusion flow^23^. Its principle was first proposed by Brace *et al*^13^. It consists of a sample connected with two reservoirs that have an equilibrated initial pressure P_0_ in the whole system. An increment pressure ΔP0 is suddenly imposed into one of the two reservoirs and the pressure evolution in two reservoirs over time is recorded as demonstrated in Fig. 1a. Analyzing the variation of gas pressures, permeability can be then determined either using the method developed by Brace^13^,^21^ or back analysis method^20^.

Based on the principle of pressure transient method, a specific setup of gas permeability measurements has been designed and developed in the Institute of Rock and Soil Mechanics of Wuhan, CAS. In this system, a triaxial core holder, capable of accepting membrane-sheathed cylindrical samples (2.5 cm diameter) and of applying independent loading in the radial and axial directions, is connected to two gas reservoirs. In order to shorten the testing time, small reservoirs are designed and the volume of upstream reservoir is about 8.2 cm^3^ and the volume of downstream reservoir is about 6.6 cm^3^. Axial and confining (radial) stresses up to 56 MPa are independently applied using two ISCO 260D pumps with control up to ± 0.007 MPa. Gas pressure is controlled by a ISCO 500D pump. Different transducers (temperature, pressure, and displacement transducers) are connected to a computer to automatically record the experimental data. All parts of this apparatus are located in a temperature controlled cabinet where the temperature can be maintained constant with a deviation of +/− 0.1 °C. The whole system is shown in Fig. 1b. Tests on aluminum samples have been carried out to calibrate the system and check possible leakage and the gas permeability accuracy of the system is up to 10^−23^ m^2^.

### Sample description

**Figure 2:**
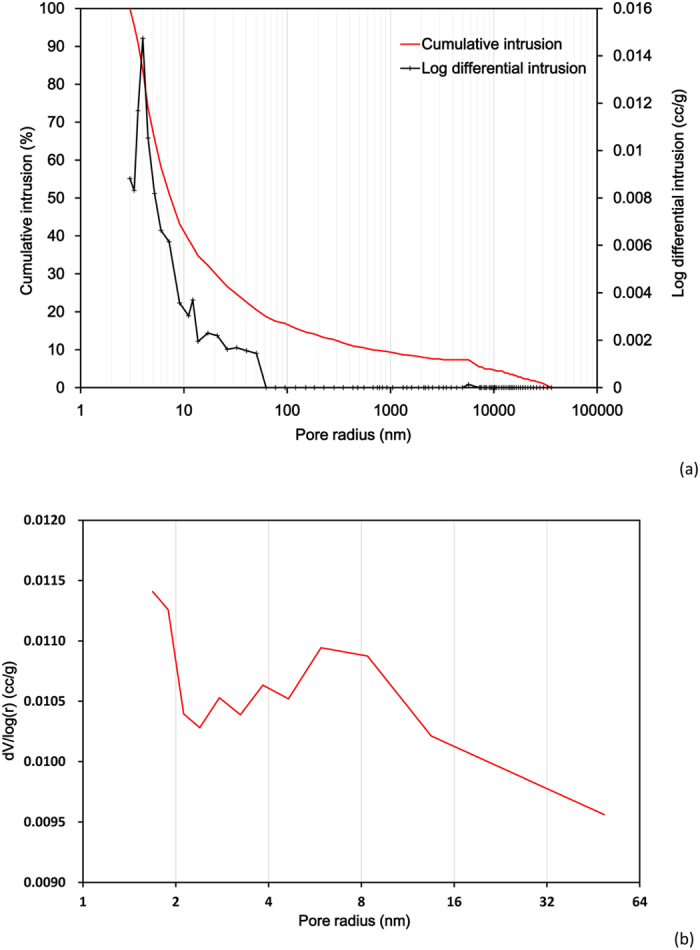
Pore size distribution of Longmaxi shale using (**a**) the MIP method and (**b**) the gas adsorption method.

In this study, shale samples were recovered from the Lower Silurian Longmaxi (LM) shale formation located in Qianjiang, southeast of Chongqing. Longmaxi shale formation is comprised of dark gray to black graptolite shale, carbonaceous shale, siliceous shale, silty shale, and argillaceous siltstone^24^. The organic content of shale is larger than 0.5%, with a mean TOC of 2.54%. LM shales are mainly composed of clay, quartz and calcite, while in contrast pyrite and feldspar are minor. The grain density of shale sample is about 2.72 g/cm^3^ and the total porosity is 0.25–3.25% and the initial water content is less than 4%. Two cylindrical samples with a diameter of 25.0 mm and a length of 40.4 mm were drilled from the same LM shale block and they were undamaged and the measured longitudinal wave velocity (Vp) at the initial state was nearly the same (2267 m/s for the sample No.1 and 2254 m/s for the sample No.2). The dry density of the sample is 2.32 g/cm^3^ and the connected porosity measured by Autopore IV9510 is 2.50%. The pore size distribution of LM shale has been characterized by the mercury intrusion porosimetry (MIP) method (Fig. 2a) and by the gas adsorption method (Fig. 2b). The results show that the pore size of LM shale is complex and it covers a range from several nanometers to hundred nanometers and the average pore size is 3.0 nm.

### Experimental procedure

**Figure 3:**
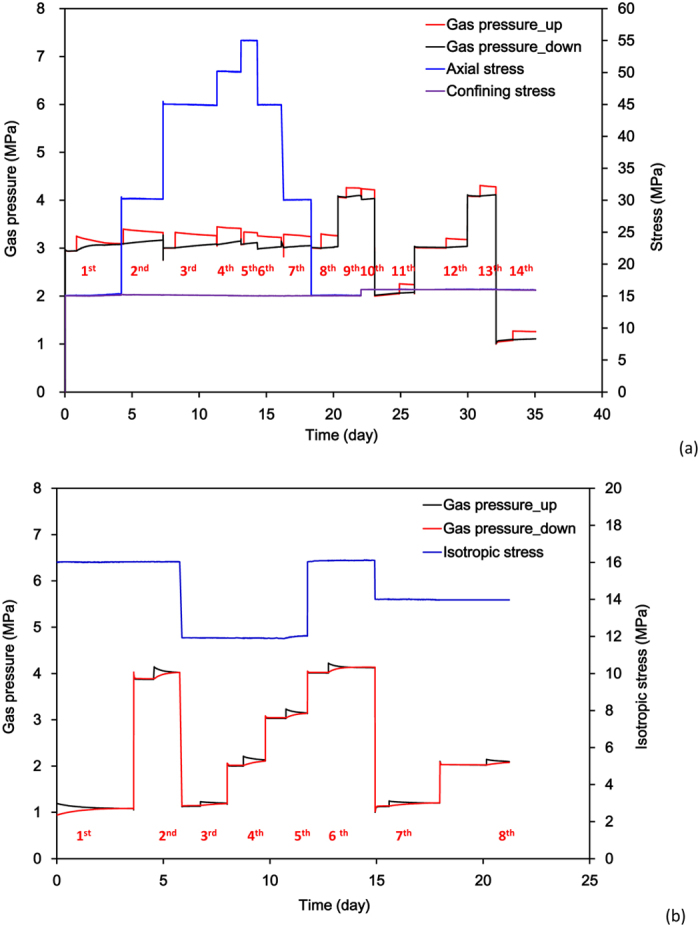
Mechanical loading path and gas pressure evolution during gas permeability tests (**a**) wet sample No.1, (**b**) dry sample No.2).

In order to evaluate the coupled effect of effective stress and gas slippage on gas permeability of shale, a series of gas permeability tests have been performed on the shale samples subjected to different confining stresses and gas pressures. Sample No.1 was at unsaturated state and the water content was 1.24%, which was measured at the end of testing by drying it at 105 °C for 24 hours. Before testing, sample No.2 with an initial water content of 1.50% was dried. To estimate the effect of effective stress on gas permeability, a cycle of loading and unloading is performed on the sample No.1 with a constant confining stress at 15 MPa. The path of applied axial stress follows: 15 MPa → 30 MPa → 45 MPa → 50 MPa → 55 MPa → 45 MPa → 30 MPa → 15 MPa. The maximum deviatoric stress is about 40 MPa, under which little damage could occur in the shale samples. At different stress levels, the initial gas pressure was maintained at 3 MPa, and the increment gas pressure for the pressure pulse varied between 0.2–0.4 MPa to measure gas permeability. At the end of the mechanical unloading, a hydrostatic stress of 16 MPa was applied on the sample No.1 and a series of gas permeability measurements were carried out with different gas pressures (1 MPa, 2 MPa, 3 MPa, 4 MPa) to study the gas slippage effect. Three levels of hydrostatic stress (12 MPa, 14 MPa, 16 MPa) were applied on the dry sample No.2 and under these stress conditions, different gas pressures were selected to measure the gas permeability of the sample. The histories of mechanical loading and gas pressure performed on the samples (No.1 and No.2) are illustrated in Fig. 3a and b, respectively. Finally, 14 pulse tests have been conducted for sample No.1 and 8 pulse tests for sample No.2 and they are shown in Fig. 3. The duration of the tests has lasted about 60 days. In this study, nitrogen was chosen to measure gas permeability and the temperature of the whole system was kept at 30 °C.

## Results

### Determination of the apparent gas permeability

The apparent gas permeability *K*_*a*_ was firstly evaluated using Brace’s equation^13^,^25^,


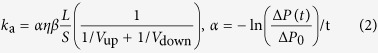


where V_up_ and V_down_ (m^3^) are the volumes in the upstream reservoir and downstream reservoir; P_up_ is the gas pressure in the upstream reservoir (Pa) and P_f_ is the final equilibrium pressure of the system (Pa); η (Pa.s) is the Nitrogen viscosity at the temperature of 30 °C and the mean pore pressure; β is the isothermal compressibility coefficient of the pore fluid (Pa^−1^) and it is gas pressure dependent; α is the decay exponent; L and S are the length and cross-sectional area of the sample, and t is the testing time (s).

Brace’s formula was derived assuming that Darcy law is valid and gas is ideal gas. The fluid volume in the pores of the rock sample is ignored. However, this will not induce a large error for tight rock with very low porosity (e.g., granite, shale). To estimate the error, a series of permeabilities, chosen by changing the value determined from the formula (2) with an increment of several 10^−22^ m^2^, were used to calculate the theoretical pressure in the upstream and downstream reservoirs following Brace’s method as the following.


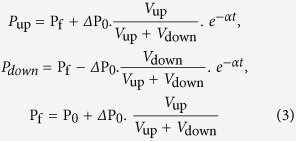


**Figure 4:**
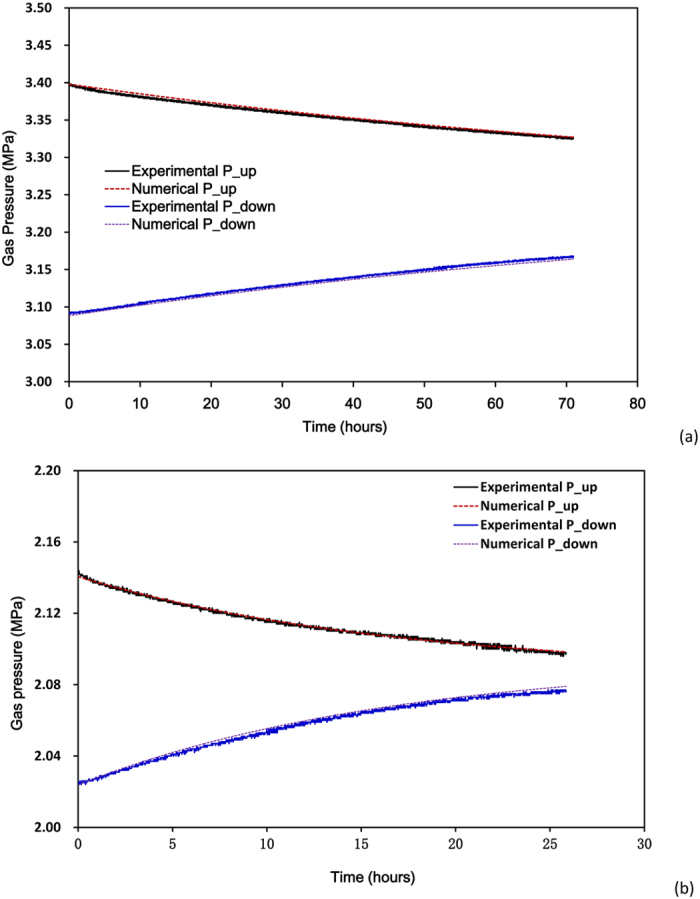
Experimental and numerical data of gas pressure evolutions in upstream and downstream reservoirs during pressure transient tests for determination of gas permeability (**a**) 2^nd^ stage of the wet sample, K_a_ = 4.3 × 10^−21^m^2^, (**b**) 8^th^ of the dry sample, K_a_ = 39.5 × 10^−21^m^2^).

Numerical results were then compared with experimental results (e.g., Fig. 4a and b). These two figures illustrate the evolution of the gas pressure in the upstream and downstream reservoirs during the pulse tests of the sample No.1 (3^rd^ stage) and No.2 (4^th^ stage), respectively. The results show that the numerical results matches well against the experimental results with an appropriate *K*_*a*_.

The obtained apparent gas permeability *K*_*a*_ and the gas permeability determined directly from Brace’s formula 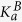 of the shale samples at different stages are listed in the Table 1 and 2. Table 1 presents the gas permeability evolution *K*_*a*_ of sample No.1 at different stages, and Table 2 lists permeabilities measured for sample No.2. The results show that the difference between the permeability determined from the formula (a) and the appropriate obtained during the numerical comparison is less than 5%. As the shale samples have a very low porosity (<2%), we believe that the Brace method is reliable in determining gas permeability in this work.

### Permeability versus deviatoric stress with the same gas pressure

As shown in Table 1, the apparent gas permeability decreases during the loading process and increases during the unloading process, but the permeability is not fully recoverable. The reduction of permeability can be explained by the compression of the shale due to the increase of stress which will reduce the effective porosity. At the first stage of loading, during which the deviatoric stress increases to 15 MPa, permeability change (from 17.6 × 10^−21^ m^2^ to 4.3 × 10^−21^ m^2^) is much larger compared with other stages during which the variation is less than 1 × 10^−21^ m^2^. This could be due to the fact that the first stage is the hydrostatic loading while the following stages are all deviatoric loading. Permeability change is consistent with the deformation evolution of the sample. The axial strain obtained during the hydrostatic loading is larger than that during the deviatoric loading for the same magnitude variation of stresses. Permeability decline is consistent with the compression of the sample during mechanical loading. The irreversible permeability loss is believed to be related to the irreversible strain, which is 0.18% in the axial direction at the end of the cycle of loading and unloading.

### Permeability versus gas pressure under constant hydrostatic stress

**Figure 5:**
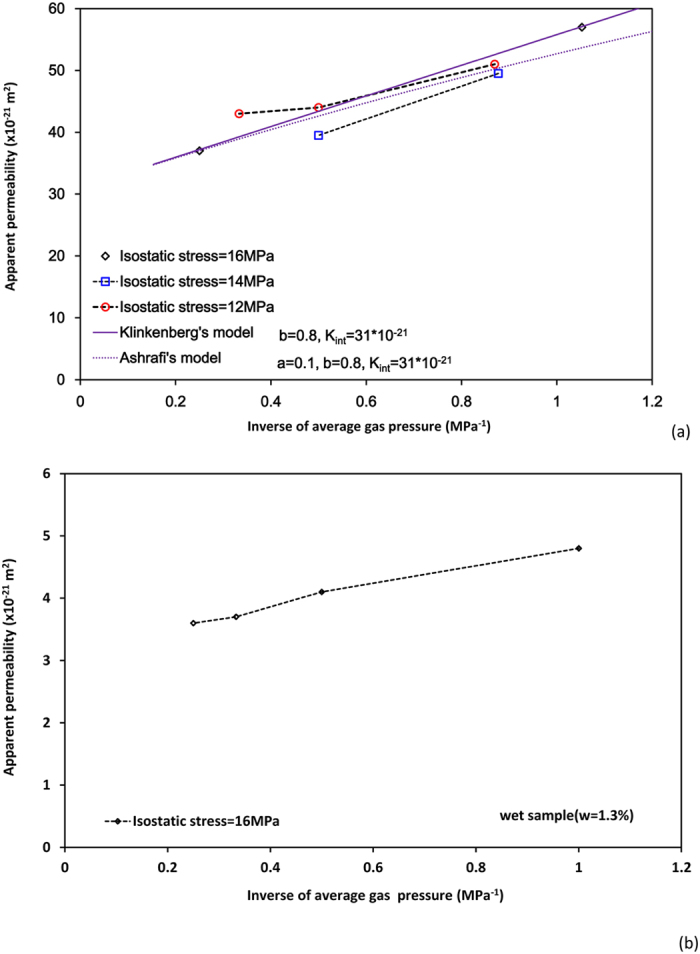
Permeability versus gas pressure at different isotropic stress (**a**) wet sample No.1, (**b**) dry sample No.2).

The results in Table 1 and 2 demonstrate that measured permeability of the samples (No.1 and No.2) decreases with the increase of gas pressure under different constant hydrostatic stresses (Fig. 5) below a certain pressure value. This permeability reduction is often explained by the effect of gas slippage which is weakened when the gas pressure increases. Sample No.2 was subjected to an isostatic stress of 16 MPa, 12 MPa, 14 MPa, successively. For the same level of gas pressure (about 1 MPa), permeability is smaller at the isostatic of 14 MPa than at 12 MPa. This should be related to the dependence of permeability on the stress as presented above. The higher stress induces a large compression of shale and thus reduces the effective porosity and results in a decrease in permeability. Permeability of sample No.2 is much larger than of sample No.1 under similar conditions, which can be explained by the role of water saturation on gas permeability.

## Discussions

Our experimental results show that measured apparent permeability is influenced by both confining stress and gas slippage effect. The effective stress principle by Terzaghi is widely used to characterize the influence of pore pressure on the material. Biot’s effective stress 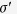
can be defined by 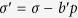, where 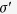 is the Cauchy stress tensor, p is pore pressure and b’ is Biot’s coefficient. In linear elastic poromechanics^26^,^27^, it is calculated by b’ = 1 − K/K_s_, where K_s_ is the bulk modulus of solid grain, and K is the apparent elastic bulk modulus under drained conditions. For soft material such as sand and soils, K«Ks, and then the Terzaghi effective stress is recovered since b’ = 1. For hard material, b’ is often less than 1. Bemer *et al*.^28^ identified Cox shale Biot’s coefficient and obtained an average value equal to 0.52. Homand *et al*. found that Biot’s coefficient decreased from 0.95 to 0.55 when the axial stress increased from 8 to 24 MPa^20^. Cariou *et al*.^29^ characterized Biot’s coefficient of partially saturated Cox shale and identified the apparent Biot’s coefficient as 1. In this study, Biot’s coefficient is evaluated by analyzing the difference in axial strain induced by confining stress and by gas pressure. For the wet sample No.1, the axial strain is increased to 0.49% when the hydrostatic stress increases to 15 MPa. It is about −0.017% when the gas pressure increases to 2.9 MPa. For the dry sample No.2, the axial strain variation is about 0.009%/MPa during gas pressure increasing period and 0.024%/MPa during hydrostatic stress loading period. The different contribution of hydrostatic stress and gas pressure on the axial strain shows that Biot’s coefficient of LM shale is less than 1.These displacement measurements confirm that different gas pressures will induce a non-negligible strain. The augment of effective stress, which is controlled by confining stress and gas pressure, will compact the pore volume and result in a decrease in permeability. Permeability decreases from 44×10^−21^ m^2^ to 39.5×10^−21^ m^2^ when the hydrostatic stress increases from 12 MPa to 14 MPa. Under the same hydrostatic stress, increasing gas pressure will expand the pores and result in an increase in permeability, which is the opposite effect compared with the influence of gas slippage. The relationship between permeability and gas pressure is nonlinear (Fig. 5) and the Klinkenberg empirical formula cannot be directly used to characterize the intrinsic permeability of the Longmaxi shale used in this work. We posit this would hold for other material sensible to the pore pressure change as well. Therefore, the influence of the effective stress on permeability cannot be ignored, and it is suggested to be considered to expand the Klinkenberg’s model.

Increasing gas pressure reduces effective stress, and also shortens the mean free path of gas molecules and thus limits the effect of the gas slippage phenomenon. The mean free path of the gas is defined as


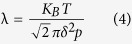


where *K*_*B*_ is the Boltzmann constant (*K*_*B*_ = 1.3805 × 10^−23^J/*K*), δ is the collision diameter of the gas molecule (for nitrogen, 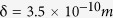), T is temperature (K), and P is the pressure (Pa). When gas pressure is 1 MPa, 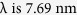 and it becomes 1.92 nm when gas pressure increases to 4 MPa. The mean free path of the gas is comparable to the average pore size of Longmaxi shale. As presented in the sample description section, the average pore size of LM shale is about 3.0 nm. The ratio of mean free path of the gas (λ) to the average pore diameter of material (d) is named as the Knudsen number 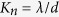. Different Knudsen number means different gas flow regimes in material^3^: continuum flow for *K*_*n*_ < 0.001, slip flow (0.001 < *K*_*n*_ < 0.1); transition flow (0.1 < *K*_*n*_ < 10) and free-molecule flow (*K*_*n*_ > 10). As shown in the MIP curve (Fig. 2), the pore size distribution of Longmaxi shale is wide thus a variety of flow types might occur in Longmaxi shale, similar to those observed by Dadmohammadi *et al*.^22^,^23^. As pores become smaller, gas slippage phenomenon will be more significant and Darcy’s law will be invalid. This phenomenon is often evaluated by Klinkenberg’s equation (Eq. 1). This equation establishes a linear relationship between the apparent permeability and the inverse of average pressure (1/p_m_). However, our experimental results (Fig. 5) cannot be explained by Klinkenberg’s linear equation. With increasing gas pressure, we also observe a pressure threshold at which the dominant effect on the net permeability changes from gas slippage to effective stress.

Based on Klinkenberg’s work, Ashrafi *et al*. proposed a new expanded equation^4^,


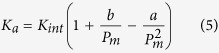


This model describes a quadratic dependency between apparent gas permeability and mean pressure. The constants, *a* and *b* are dependent on the fluid properties and pore geometry, *b* is similar to the Klinkenberg constant and *a* is the slip effect with a non-constant velocity distribution and could be a representation of a dynamic or secondary slippage factor. This new equation can describe the nonlinear relationship between the apparent permeability and 1/p. However, this equation cannot completely explain the apparent permeability evolution. The obtained data in this work were used to fitting Klinkenberg and Ashrafi’s models and the results were shown in Fig. 5 which shows permeability change is different from the theoretical predictions from Klinkenberg and Ashrafi’s model. Specifically, when gas pressure increases to a certain threshold, net permeability increases again, and this is dominated by the effective stress principle. Moreover, both Klinkenberg’s equation and Ashrafi’s model consider that the pore size is constant while gas pressure increases. This is certainly not the case for geomaterial subjected to mechanical loading. Therefore, one needs to consider the coupled effect of effective stress and gas slippage in order to obtain an accurate estimation of permeability.

The exponential relationship^30^ is often used to express the dependence of the permeability on the effective stress and it is adopted as follows


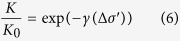


where *K* is the permeability of the material subjected to the effective stress change (m^2^), *K*_0_ is the initial permeability of the material (m^2^), *γ* is a material property (Pa^−1^), 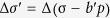 is the effective stress change. This model can represent the increase of permeability due to the reduction in effective stress. To expand the Klinkenberg’s model to incorporate both gas slippage and effective stress effects, we propose a new conceptual model expressed as,


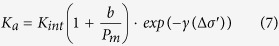


This model is capable of replicating the coupled and competing effect of both gas slippage effect and effective stress effect. It allows the dominant effect on the net permeability to switch from gas slippage to effective stress when increasing gas pressure. More tests will be performed in our future research activities to identify these parameters and better constraint this conceptual model.

The measured gas permeability of the wet sample No.1 is lower than that of the dry sample No.2. Given that the porosity of LM shale is 2.5% and the water content is 1.46%, the degree of water saturation of sample No.1 is estimated to be 83%. The dependence of gas permeability on water content has been observed in the past on Cox shale^25^. The experimental results of Yang *et al*.^25^ show that there exists a quasi-linear relationship between log(*K*_*a*_) and the saturation. The obtained results in this work confirm that permeability of shale strongly depends on water content and it should be considered during gas production design, because gas and water often co-exist in shale formations. It is necessary to further characterize the gas permeability of unsaturated shale in the future, although it would be challenging in the laboratory to control the saturation of the sample to as the porosity of Longmaxi shale (2.50%) is much less than that of Cox shale (18%).

In this study, nitrogen is used to measure gas permeability and it is a slightly sorptive gas which could induce swelling of shale. Nuttall *et al*. studied the adsorption of Devonian black shales and found CO_2_ have an adsorption capacity approximately 5 times greater than that of CH_4_^31^. Battistuta *et al*.^32^ and Wang *et al*.^17^ studied the sorptive effect of different gas (CO_2_, CH_4_ and N_2_) on dry coal and found that CO_2_ sorption on coal induces a bigger swelling effect on the coal matrix than CH_4_ and N_2_. Wu and Zhang^33^ found that the swelling of matrix decreases gas permeability. In this study, the adsorption effect on permeability was checked by repeated gas permeability tests under the same condition at different stages as shown in Fig. 3a,b and Tables 1, 2 and the results show that permeability does not change even though the gas tests have lasted two weeks. The reason could be due to the fact that N_2_ has a relatively weak adsorption capacity. Thus, the swelling of shale due to gas adsorption can be ignored to characterize gas permeability in this study.

## Additional Information

**How to cite this article:** Yang, D. *et al*. Experimental investigation on the coupled effect of effective stress and gas slippage on the permeability of shale. *Sci. Rep.* **7**, 44696; doi: 10.1038/srep44696 (2017).

**Publisher's note:** Springer Nature remains neutral with regard to jurisdictional claims in published maps and institutional affiliations.

## Figures and Tables

**Table 1:** Apparent permeability of shale sample No.1 at different stages.

Stage in Fig. 3a	1^st^	2^nd^	3^rd^	4^th^	5^th^	6^th^	7^th^	8^th^	9^th^	10^th^	11^th^	12^th^	13^th^	14^th^
Confining stress (MPa)	15	15	15	15	15	15	15	15	15	16	16	16	16	16
Axial stress (MPa)	15	30	45	50	55	45	30	15	15	16	16	16	16	16
Gas pressure (MPa)	3	3	3	3	3	3	3	3	4	4	2	3	4	1
K_a_ (x10^−21^ m^2^)	17.6	4.3	3.9	3.7	3	3.1	3.5	3.7	3.6	3.6	4.1	3.7	3.6	4.8
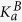 (x10^−21^ m^2^)	17.1	4.4	3.9	3.6	3.1	3.2	3.5	3.6	3.5	3.5	4.1	3.6	3.5	4.7

**Table 2:** Apparent permeability of shale sample No.2 under the constant hydrostatic stress for different gas pressures.

Stage in Fig. 3b	1^st^	2^nd^	3^rd^	4^th^	5^th^	6^th^	7^th^	8^th^
Hydrostatic stress (MPa)	16	16	12	12	12	16	14	14
Gas pressure (MPa)	1	4	1.15	2	3	4	1.14	2
K_a_ (x10^−21^ m^2^)	57	37	51	44	43	37	49.5	39.5
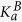 (x10^−21^ m^2^)	56.8	37.2	50.9	44.2	43.1	36.9	49.8	39.7
